# Upregulation of LncRNA PVT1 Facilitates Pancreatic Ductal Adenocarcinoma Cell Progression and Glycolysis by Regulating MiR-519d-3p and HIF-1A

**DOI:** 10.7150/jca.37959

**Published:** 2020-02-14

**Authors:** Junwei Sun, Pingping Zhang, Tao Yin, Feng Zhang, Weixing Wang

**Affiliations:** 1 Department of Hepatobiliary and Laparoscopic Surgery, Renmin Hospital of Wuhan University, 238 Jiefang Road, Wuhan, Hubei 430060, China.; 2 Department of Radiation Oncology, Hubei Cancer Hospital, Affiliated Hubei Cancer Hospital of Huazhong University of Science and Technology, 116 Zhuodaoquan South Road, Wuhan, Hubei 430079, China.; 3 Department of Hepatic & Biliary & Pancreatic Surgery, Hubei Cancer Hospital, Affiliated Hubei Cancer Hospital of Huazhong University of Science and Technology, 116 Zhuodaoquan South Road, Wuhan, Hubei 430079, China.

**Keywords:** PDAC, PVT1, miR-519d-3p, HIF-1A

## Abstract

The long, noncoding RNA (lncRNA) PVT1, as an important epigenetic regulator, has a critical role in carcinogenesis. However, its role in pancreatic ductal adenocarcinoma (PDAC) has not been fully investigated. Here, the up-regulated expression of lncRNA PVT1 is found in our PDAC tumor samples. Knockdown of it suppressed PDCA cells growth and glycolysis. An inverse association between miR-519d-3p and PVT1 was found. RIP, RNA pulldown and luciferase assay showed that PVT1 directly targets miR-519d-3p by binding with microRNA binding site. Bioinformatics analysis and study indicated that HIF-1A is a target of miR-519d-3p. Collectively, our findings suggested that PVT1 could act as an oncogenic lncRNA, and promote tumor progression by regulating HIF-1A via competing with miR-519d-3p.

## Introduction

Pancreatic ductal adenocarcinoma (PDAC) is one of the most aggressive cancers with very poor prognosis despite recent advances in treatments 1. The exact molecular mechanisms of PDAC still need to be explored. Understanding the molecular mechanism of PDAC could be helpful to identify novel therapies.

Long noncoding RNAs (lncRNAs), as a class of functional RNA transcripts at 200 to 100,000 nt, have been implicated many human diseases^2^. Recent studies showed that lncRNAs could act as molecular sponges to compete microRNAs (miRNAs) 3.

Glucose metabolism is re-programmed in cancer cells: the consumption of glucose through glycolysis pathway is increased 4. HIF-1 is crucial in the glucose metabolism, proliferation, and invasion of cancer 5. These suggest that lncRNAs and/or miRNAs may regulate glycometabolism, proliferation, and invasion of cancer by HIF-1.

Recent studies have shown that amplification of the lncRNA plasmacytoma variant translocation 1 (PVT1) contributes to the pathophysiology of cancers, including pancreatic cancer 6, 7, 8, 9. Increased expression of the lncRNA PVT1 in cancer tissue is associated with poor prognosis in pancreatic cancer patients 10. The salivary levels of PVT1 were significantly higher in PDAC group^11^. Downstream genes containing binding site of miR-519d-3p in 3'UTR of PVT-1 were analyzed by bioinformatics and previous reports^12^. miR-519d-3p has been found to be a tumor repressor in colorectal and breast cancers 13, 14. But, the role of miR-519d-3p in PDAC is still unclear. PVT1 has been reported to sponge miR-519d-3p to facilitate invasion and proliferation in laryngeal squamous cell cancer^15^. However, the mechanism of PVT1 by regulating the miR-519d-3p/ HIF-1 in cell metabolism remains largely unknown.

Here, we showed that miR-519d-3p could suppress HIF-1, as a target of miR-519d-3p. Our findings also suggest that lncRNA PVT1 directly binds to, and inhibit miR-519d-3p. Mechanistically, we speculated that PVT1 binds to, and inhibit miR-519d-3p, which in turn repress the expression of HIF-1, which in result promotes glycolysis, proliferation, and invasion of PDAC cells.

## Methods

### Ethics Statement and Clinical Sample Collection

From 2017 to 2018, 30 PDAC patients which could be simultaneously obtained the tissue samples and the adjacent noncancerous tissue samples surgically were enrolled in the current research at Hubei Cancer Hospital (Wuhan, China). All participants provided written informed consent prior to enrollment. None of the patients received any pre-operative treatments prior to sample collection. All patients' pathology results were confirmed by pathology after operation. Normal tissues were taken from the same patient at >1 cm from the edge of the primary tumor. Fresh samples were snap-frozen in liquid nitrogen and stored at -80°C prior to RNA isolation. Hubei Cancer Hospital Ethics Committees approved this study([2018]KYLL-07).

### Cell culture

Human pancreatic adenocarcinoma cells (HPAC, DANG, BXPC3, PANC1, and ASPC-1) and H6C7 cells were obtained from the Chinese Academy of Sciences (Shanghai, China). Cells were cultured in DMEM high-glucose medium (Gibco, USA) in a humidified incubator containing 5% CO_2_.

### Quantitative real-time PCR (qRT-PCR) analysis

Small interfering RNAs (siRNAs) against lncRNA PVT1 were purchased from Ribobio (Guangzhou, China). MiR-519d-3p mimics and negative-control (NC) mimics were obtained from Ribobio (Guangzhou, China). The primer sequencesof the following primers: PVT1 forward: 5′ - TTG GCA CAT ACA GCC ATC AT -3′; PVT1 reverse: 5′- GCA GTA AAA GGG GAA CAC CA -3′; miR-159-3p forward: 5′- GAC CAA AGT GCC TCC CTT TA -3′; miR-159-3p reverse: 5′- CAG TGC GTG TCG TGG AGT -3′. SYBR-Green PCR kit (Takara Biotechnology, China) was used for qRT-PCR. U6 and β-actin were used as control for normalization. The relative RNA expression change was determined using 2^-△△Ct^ method.

### Vector construction and Luciferase reporter gene assay

The PVT1 cDNA was cloned into the pcDNA3.1 (Invitrogen, USA) plasmid. The 3′UTR nucleotides of lncRNA PVT1 that contains the miR-519d-3p binding target site (AGGCACUUU) was inserted into the pmirGLO vectors (pmirGLO-PVT1-wt). The mutant miR-519d-3p binding target site in 3′UTR nucleotides of PVT1 was generated by PCR mutagenesis (Stratagene, USA); the plasmid was referred to as pmirGLO-PVT1-mut. The pmirGLO-HIF-1A3′UTR-wt (containing miR-519d-3p binding domain of the 3′UTR of HIF-1A) and pmirGLO-HIF-1A3′UTR-mut were constructed based on the above methods. The PDAC cells were co-transfected with miR-519d-3p mimics/ NC mimics (40-nM), and pmirGLO-PVT1-wt/pmirGLO-PVT1-mut or pmirGLO-HIF-1A3′UTR wt/pmirGLO-HIF-1A3′UTR -mut) (30-ng). The dual luciferase assay kit (Promega) was used to measure luciferase activity.

### RNA immunoprecipitation (RIP) assay

RIP assays were performed using the EZ-Magna RIP RNA-Binding Protein Immunoprecipitation Kit (Millipore, USA). PDAC cells were lysed and then treated with RIP buffer. The co-precipitated RNAs were isolated to detect miR-519d-3p expression with qRT-PCR.

### RNA pull-down assay

PDAC cells were preincubated with biotinylated miR-519d-3p. Magnetic beads were used and then been washed. qRT-PCR was performed to measure the eluted RNA.

### MTT assay

Cells were seeded in a 96-well plate And the cells were used by MTT assay Kit(Thermo, USA) at 24, 48, and 72 h and then the absorbance measured by microplate spectrophotometer (Molecular device, USA) at 490 nm.

### Colony formation assay

Transfected PDAC cells were cultured in a special medium in six-well plate for 14 days. Cells were fixed with and stained for counting.

### Bioinformatics analysis

The binding target sites of PVT1 and miR-519d-3p were predicted using TargetScan (http://targetscan.org/) and Microrna (http://www.microrna.org/). Expression pattern of PVT1 in a cohort of PDAC patients in TCGA (https://tcga-data.nci.nih.gov/tcga).

### Western blot

HIF-1A antibody was obtained from Santa Cruz Biotechnology. LDHA, GLUT1, β-actin and HK2 antibodies were purchased from Cell Signaling. The membrane was incubated with these antibodies and then with a horseradish peroxidase-conjugated secondary antibody for 1 h. Protein concentration was visualized by enhanced chemiluminescence (Cell Signaling Technology) and determined by BCA method.

### Glucose uptake, lactate secretion and intracellular ATP Assay

Cells were preincubated with glucose-free DMEM, and then cultured in high-glucose DMEM for 24h. Glucose uptake, lactate secretion and intracellular ATP assay were examined using the Glucose Assay Kit (JianCheng, China), the Lactate Assay Kit (JianCheng) and the ATP Assay Kit (JianCheng), respectively. Experiment was repeated three times.

### Wound healing assay

Cells were incubated in plates until cells reached a 90% confluence, and then incubated for 24 h after scratching with a 200-ml tip. The mean migrating length was assessed by photography.

### Cell migration assay

Invasion assays were performed in boyden chamber. Cells were added to the upper chamber containing DMEM, while DMEM with 10% fetal bovine serum was added to the lower chamber. Cells were incubated, fixed, stained, and then counted in five fields under 20×objective magnification.

### Tumor xenograft experiment in nude mice

HPAC cells stably expressing PVT1 shRNA or its control were hypodermically injected into the dorsal flank of the athymic male BALB/c nude mouse (Shanghai SLAC Laboratory Animal Co.) (n = 6 per group). Tumors were measured. Western blot analysis and immunohistochemistry were perform. Animal experiments were approved by Hubei Cancer Hospital Ethics Committees ([2018]KYLL-07).

### Immunohistochemistry (IHC)

Tumors from nude mouse were fixed, embedded, and incubated with HIF-1A antibody (Santa Cruz Biotechnology, USA) and secondary antibodies. HIF-1A expression was visualized by the staining intensities.

### Statistical analysis

Data are expressed as the mean ± SD. Student's t-test was used to compare the continuous variables. Kaplan-Meier survival curves were compared using the log-rank test for survival analysis. *P values* < 0.05 was defined as significant. Statistical analyses were carried out using SPSS 18.0.

## Results

### PVT1 is overexpressed in human PDAC and decreases patient survival

**Figure 1:**
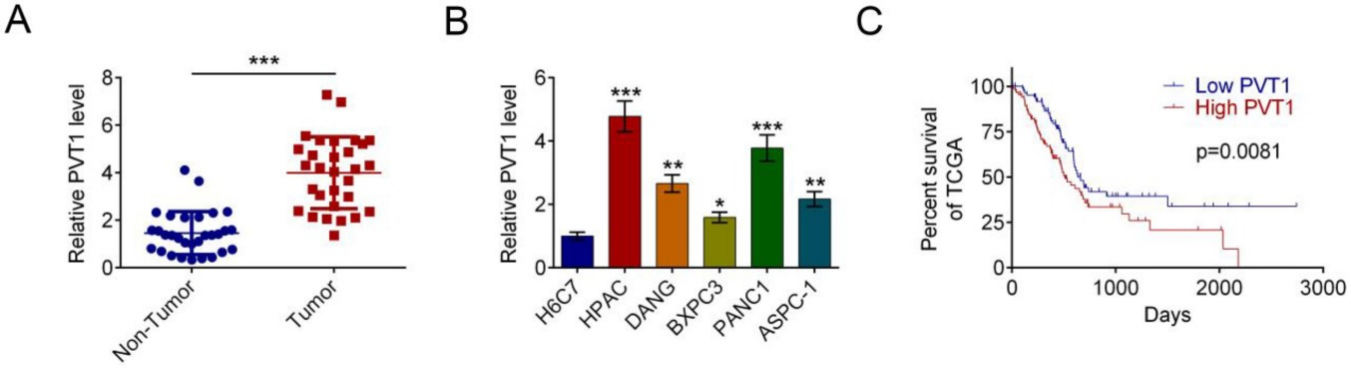
** LncRNA PVT1 is often overexpressed in PDAC cancer and correlates with poor prognosis.** (A) The level of PVT1 mRNA was measured by qRT-PCR in PDAC tissues and corresponding noncancerous tissues (n = 30). Data are indicated with medians and quartiles. (B) The level of PVT1 mRNA was measured by qRT-PCR in the normal pancreatic cell line H6C7 and various pancreatic cancer cell lines (HPAC, DANG, BXPC3, PANC1, and ASPC-1). Data are shown as the mean ± SD, n=3. (C) Expression levels of PVT1 in the TCGA cohort. Kaplan-Meier analysis of the correlation between PVT1 expression and overall survival in the TCGA cohort. The median level of PVT1 is used as the cutoff. (^*^P < 0.05, ^**^P < 0.01, ^***^P < 0.001)

qRT-PCR assay showed increased level of PVT1 in PDAC tissues in comparison with corresponding noncancerous tissues in 30 patients (Fig. 1A). We further investigated the correlation between PVT-1 mRNA expression and clinicopathologic parameters in patients (Tab. 1). High PVT-1 expression, which was defined as being above the median, correlated significantly with lymph node metastasis (*P* = 0.004). We also found higher PVT1 mRNA level in representative PDAC cell lines (HPAC, DANG, BXPC3, PANC1, and ASPC-1) vs. the human normal pancreatic ductal epithelial cell line H6C7 (P < 0.05). HPAC cells had the highest level of PVT1 mRNA among the five PDAC cell lines and, therefore, were selected for the subsequent functional experiments. Survival analysis was analyzed to found that the relation between high PVT1 level and poor survival was positive (Fig. 1C).

### Lower PVT1 inhibits the proliferation and invasion of cultured PDAC cells

**Figure 2:**
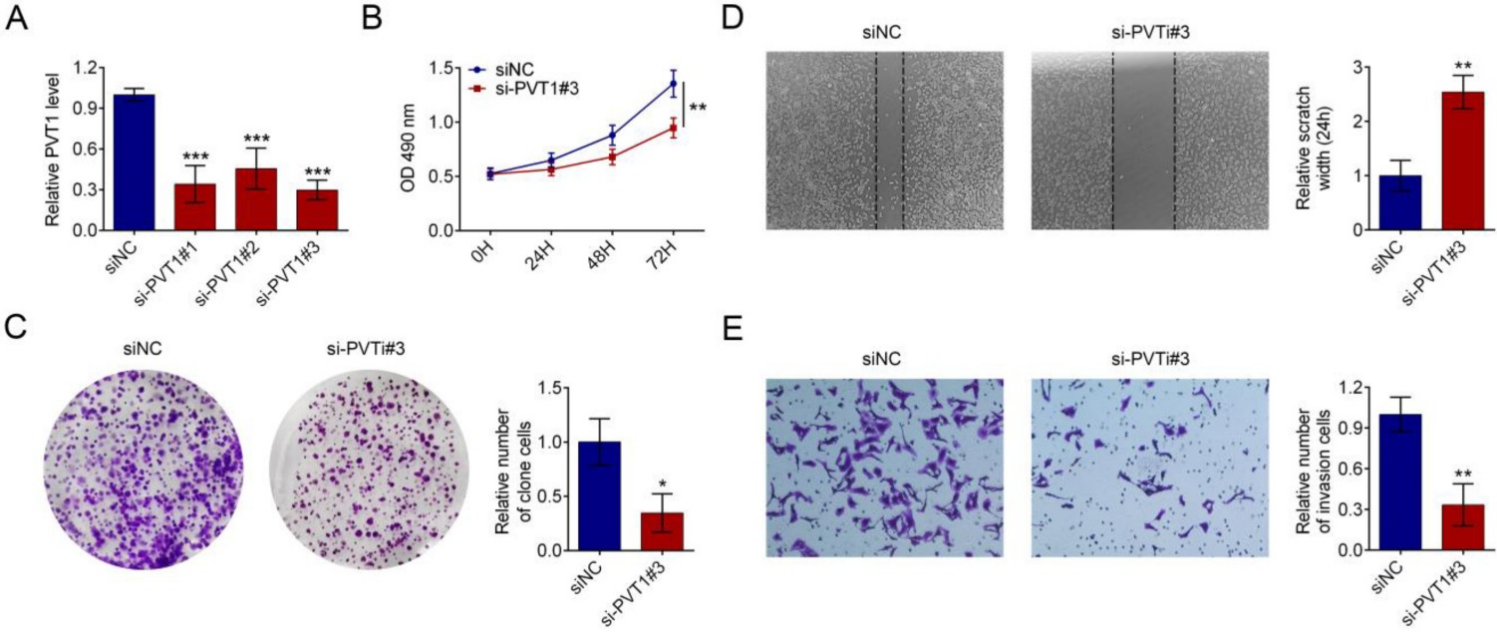
** PVT1 knockdown represses PDAC cells proliferation and invasion in *vitro*.** (A) qRT-PCR was used to measure the expression level of PVT1 in HPAC cells that had been transfected with siRNAs against PVT1 or control. (B) Cell viability was determined by MTT assay in PDAC cells transfected with control or si-PVT1#3. (C) A colony formation assay was performed to assess cell proliferation in HPAC cells transfected with control or si-PVT1#3. (D) Representative images of wound healing assays performed using PDAC cells after PVT1 was silenced for 24 h. Magnification 200×, Scale bars = 10 μm. (E) A Transwell assay was performed to assess the invasion of HPAC cells transfected with control or si-PVT1#3. All the results were reproducible in three independent experiments. Data are shown as the mean ± SD, n=3. (^*^P < 0.05, ^**^P < 0.01, ^***^P < 0.001)

HPAC cells were transfected with 3 siRNAs against PVT1 or a control siRNA. qRT-PCR assay showed that si-PVT1#3 was the most effective siRNA (Fig. 2A). Therefore, it was used for subsequent experiments. MTT assay indicated that knock-down PVT1 has an inhibitory effect on the proliferation of PDAC cells (Fig. 2B). similarly, colony formation (Fig. 2C), migration (Fig. 2D) and invasion (Fig. 2E) were reduced by PVT1 knockdown.

### LncRNA PVT1 knockdown decreases glycolysis of PDAC cells

**Figure 3:**
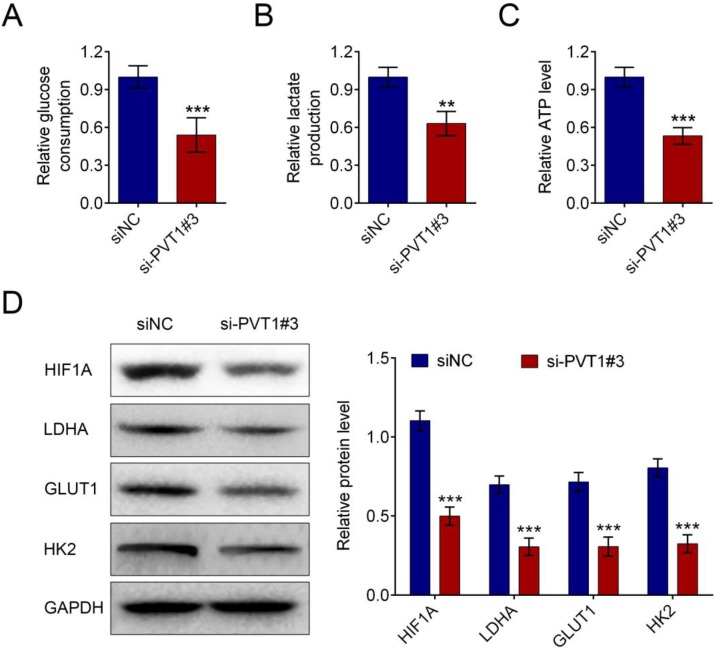
** Knockdown of lncRNA PVT1 minimized glycolysis of PDAC cells.** PDAC cells were cultured for 24 h after 48 h of transfection with either control or si-PVT1#3. Glucose uptake (A), lactate secretion (B) and intracellular ATP levels (C) of these cells were quantified and normalized for cell numbers. Shown data are mean ± SD (n = 3). Cell lysates were then analyzed by western blotting by anti-HIF-1A, anti-LDHA, anti-GULT1, anti-HK2 or anti-β-actin antibodies (D). (^**^P < 0.01, ^***^P < 0.001)

LncRNA-PVT1 knockdown decreased glucose uptake in pancreatic cancer cells (Fig. 3A). LncRNA-PVT1 knockdown also decreased lactate secretion (Fig. 3B), intracellular ATP levels (Fig. 3C), and HIF-1A, LDHA, GLUT1 and HK2 expression (Fig. 3D).

### PVT1 negatively controls miR-159-3p in PDAC cells

**Figure 4:**
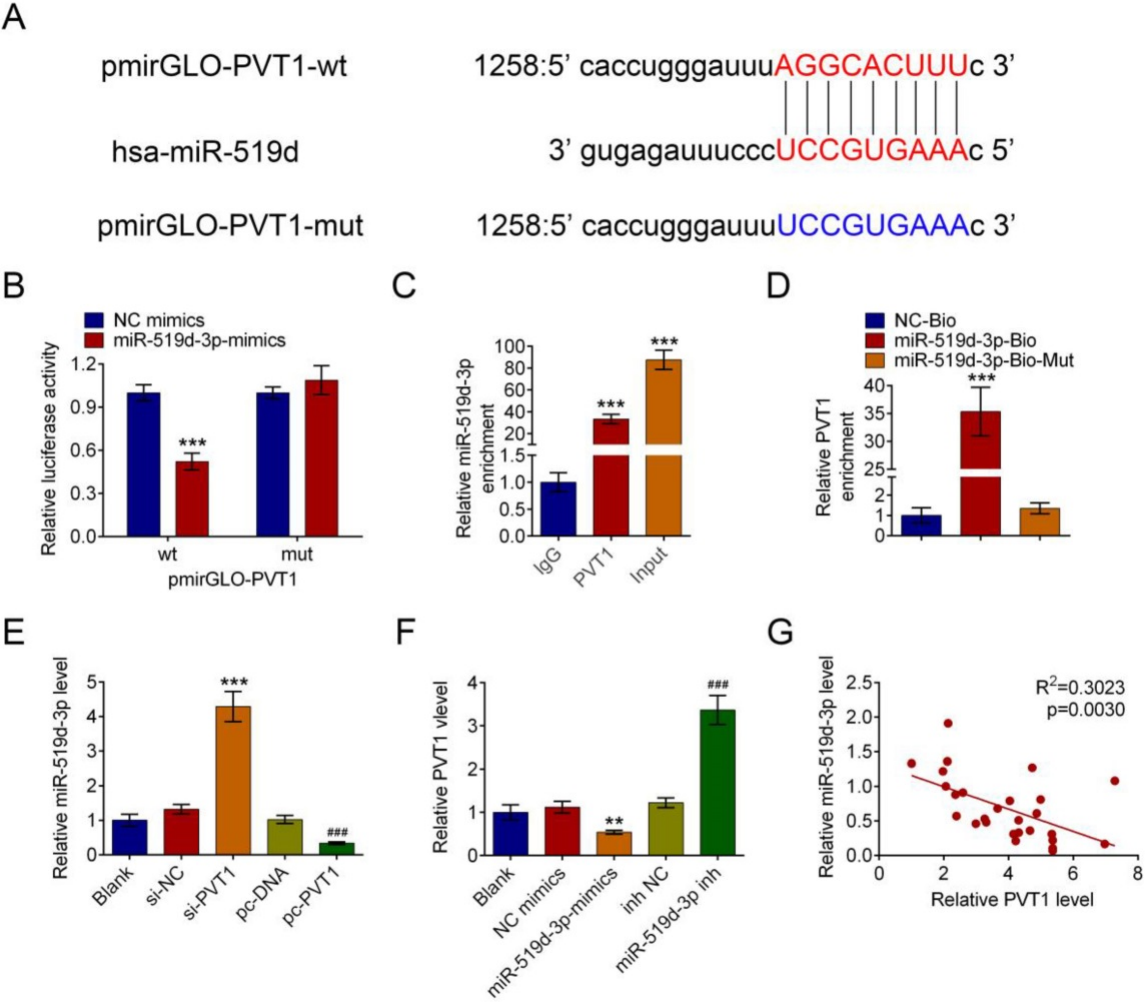
**PVT1 negatively regulates miR-159-3p in PDAC cells.** (A) The putative binding site of miR-159-3p and PVT1 was predicted by microrna.org. (B) The luciferase activity of PVT1 was detected using the luciferase report gene assay in HPAC cells co-transfected the wild type (pmirGLO-PVT1-wt) or mutated reporter construct (pmirGLO-PVT1-mut) and miR-159-3p-mimics or NC mimics. (C) Cellular lysates from HPAC cells were used for RIP with an Ago2 antibody and IgG antibody. The levels of miR-159-3p were detected by qRT-PCR. (D) HPAC cells were transfected with biotinylated NC (NC-Bio), biotinylated wild-type miR-519d-3p (miR-519d-3p-Bio) or biotinylated mutant miR-138 (miR-519d-3p-Bio-Mut), and biotin-based miRNA pull-down assays were conducted after 48 h of transfection. The PVT1 were analysed by qRT-PCR. (E) The miR-159-3p were detected in PDAC cells transfected with si-NC, si-PVT1#3, pc-DNA3.1 or pc-DNA3.1-PVT1. The PVT1 were detected in PDAC cells transfected with NC-mimics, miR-159-3p-mimics, inh NC or miR-159-3p-inh. (F) Correlation between PVT1 and miR-159-3p were detected in the cancer tissue of PDAC patients. (** and ## *P* < 0.01, ***and ### *P* < 0.001)

MiR-519d-3p was discovered to be one of the predicted downstream targets of PVT1 by bioinformatics screening(Fig. 4A). Recombinant pmirGLO-PVT1 and pmirGLO-PVT1-Mut were constructed. We found that the decrease of luciferase activity was due to the interaction between miR-519d-3p and PVT1. Meanwhile, the pmirGLO-PVT1-Mut was obviously refractory to miR-519d-3p-mediated luciferase activity repression (Fig. 4B). RNA immunoprecipitation (RIP) experiment was conducted using an Ago2 antibody. It has showed preferential enrichment of PVT1 and miR-519d-3p in the Ago2-containing miRNPs vs. the IgG control (Fig. 4C). The results showed that PVT1 could be pulled down by miR-519d-3p (Fig. 4D).

Next, the expression of miR-519d-3p was significantly increased by si-PVT1 and decreased by pcDNA-PVT1 compared with their controls (Fig. 4E). The negative correlation between PVT1 and miR-519d-3p was explored in HPAC cells. qRT-PCR in human PDAC tissue samples revealed downregulation of miR-519d-3p and negative correlation between miR-519d-3p and PVT1 levels (r^2^ = 0.3023, P = 0.0030, Fig. 4F).

### PVT1 positively relates HIF-1A, a target of miR-519d-3p

**Figure 5:**
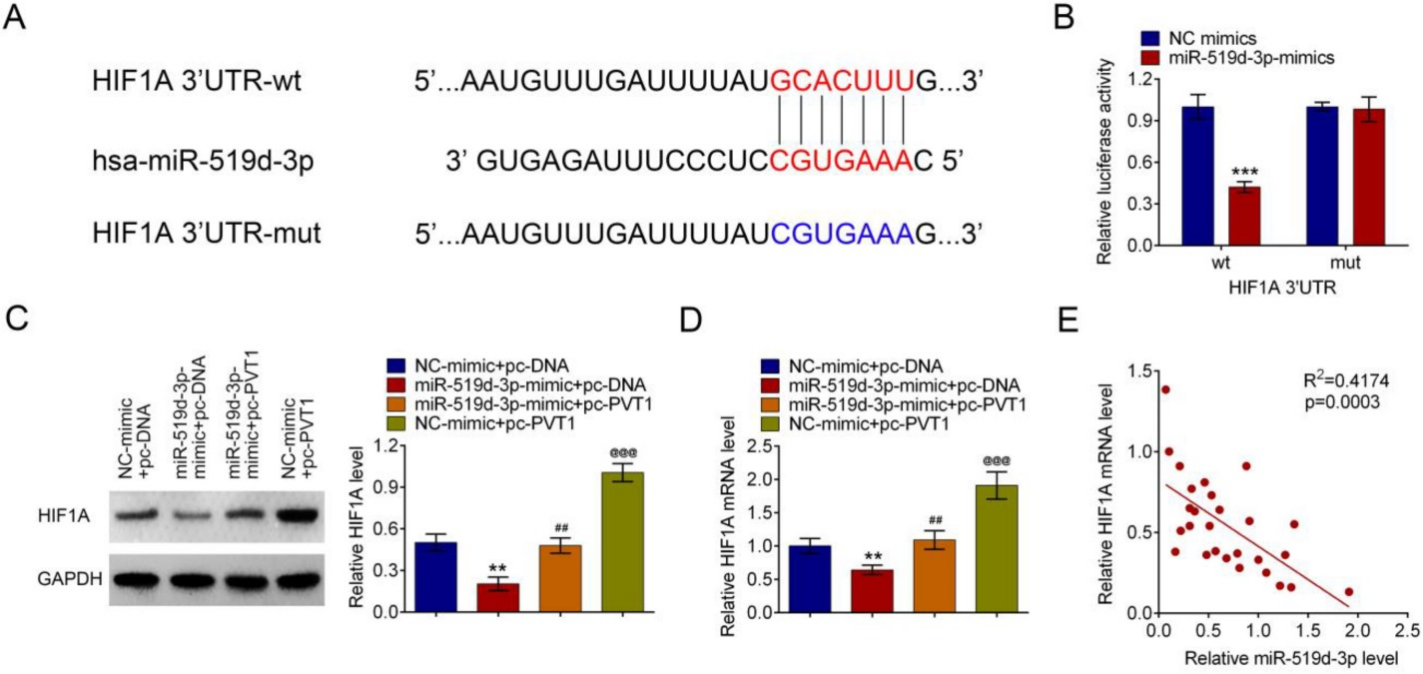
** PVT1 inhibits miR-159-3p and then positively regulates HIF-1A.** (A) Bioinformatics analysis showed the prediction for miR-159-3p binding sites on HIF-1A. (B) The luciferase reporter constructs containing the wildtype (HIF-1A3'UTR-WT) or mutant HIF-1A (HIF-1A3'UTR-MUT) sequence. HIF-1A3'UTR-WT or HIF-1A3'UTR-MUT was co-transfected with miR-159-3p mimics or NC mimics for 48 hours. (C and D) The mRNA and protein levels of HIF-1A transfected with miR-519d-3p-mimic, pc-PVT1 and their controls were measured by qRT-PCR and western blot assays. (E) Correlation analysis between HIF-1A and miR-159-3p were detected in the cancer tissue of PDAC patients. (** and ## *P* < 0.01, ***, ### and @@@*P* < 0.001))

Bioinformatics analysis suggested that HIF-1A is a target gene of miR-519d-3p. The predicted binding region between 3'UTR of HIF-1A mRNA and miR-519d-3p was detected by bioinformatics analysis (Fig. 5A). MiR-519d-3p down regulated luciferase expression in the wild-type, but not the mutant control (Fig. 5B). Amplification of PVT1 in HPAC cells increased the HIF-1A mRNA and protein expression; such an effect was reversed by the miR-519d-3p-mimic (Fig. 5C and 5D). A correlation analysis showed that HIF-1A mRNA was negatively related with the miR-519d-3p levels in human PDAC tissue samples(Fig. 5E).

### PVT1 and miR-159-3p regulate PDAC progress *in vivo* and *in vitro*

**Figure 6:**
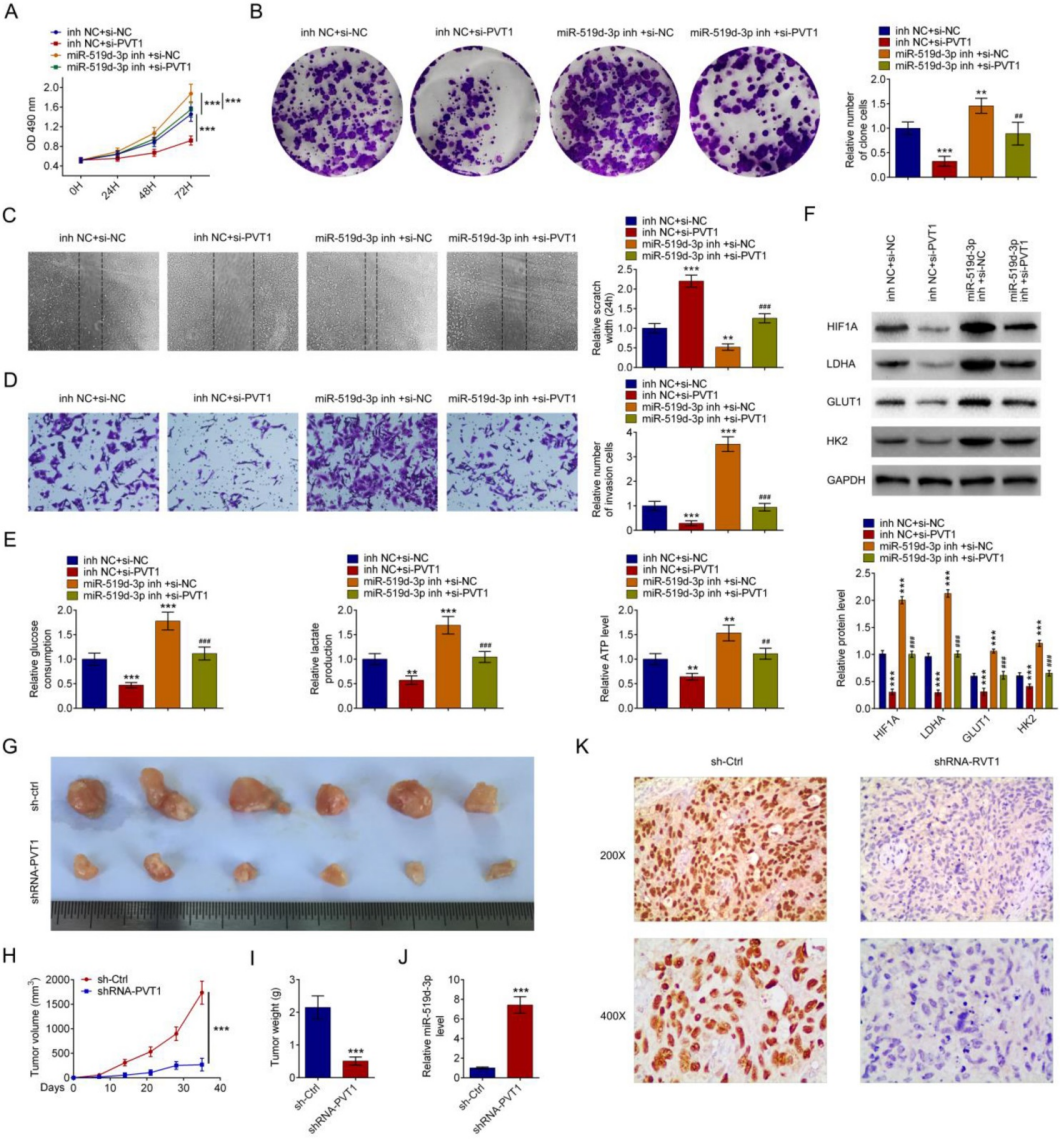
** The effect of PVT1 on progression and glycolysis is miR-159-3p dependent.** Repression of miR-159-3p overcame the inhibitory effects of decreasing PVT1 on cell proliferation by the MTT assay and the colony formation assay and on cell migration by transwell assays and wound healing assay (A, B, C and D). (E) PDAC cells were cultured for 24 h after 48 h of transfection with si-NC, si-PVT1, inh NC or miR-159-3p-inh. Glucose uptake, lactate secretion and intracellular ATP levels were quantified and normalized for cell numbers. (F) Cell lysates were then analyzed by western blotting by anti-HIF-1A, anti-LDHA, anti-GULT1, anti-HK2 or anti-β-actin antibodies. (G, H, I, J and K) A total of 2*10^6^ cells after 48 h of transfection with shRNA or PVT1 shRNA for 48h, were then injected subcutaneously into nude mice. Tumor growth curves were measured after injection (n = 6 for each group). Tumor weights were measured after the tumors were removed. The PVT1 and miR-159-3p were detected by RT-PCR. Immunohistochemistry detection indicated HIF-1A. All values are presented as mean ± SD. (** and ##* P* < 0.01, ***and ###* P* < 0.001)

miR-159-3p-inh attenuated the increase of PDAC cell proliferation caused by si-PVT1 (Fig. 6A and 6B). Wound healing assays and transwell indicated that the inhibitory effects of si-PVT1 were attenuated by miR-519d-3p knockdown (Fig. 6C and 6D). miR-519d-3p knowckdown attenuated siPVT1-indcued decrease in glucose uptake, lactate secretion, intracellular ATP levels, as well as siPVT1-induced downregulation of HIF-1A, LDHA, GLUT1 and HK2 expression (Fig. 6E and 6F). Upon transplantation in nude mice, HPAC cells transfected with shRNA-PVT1 grew at a significantly lower rate than the sh-Ctrl (Fig. 6G, 6H and 6I). qRT-PCR analysis confirmed reduced level of miR-159-3p in the shRNA-PVT1 group (Fig. 6J). Immunostaining revealed decreased HIF-1 expression in the shRNA-PVT1 group (Fig. 6K).

## Discussion

lncRNAs play important roles in gene expression, chromosome remodeling, and metabolism 16. PVT1 expression is upregulated in most human cancers, with an exception of decreased expression in thyroid carcinoma 8. LncRNA PVT1 is abnormally upregulated in most cancers, including gastric cancer, non-small cell lung cancer (NSCLC) and hepatocellular carcinoma, and has been found to be an oncogenic lncRNA 6, 7, 17. Recurrent translocations of PVT1 that arise through chromothripsis, such as PVT1-MYC and PVT1-NDRG1, often occur in malignant tumor 18. Correlation analysis further revealed that the majority of lncRNA and mRNAs were negatively correlated, suggesting that lncRNAs were likely to serve as negative regulators of their targeting coding genes in PDAC tissues^19^. In addition, recent reports showed that PVT1 can be as a competing endogenous RNA to sponge miRNAs to governs cancer characters 6, 9, 20,23.

Previous reports have reported that PVT1 promotes proliferation, cyto-protective autophagy, chemosensitivity, and migration of PDAC cells and reprogrames glucose metabolism in osteosarcoma 9, 20,21,22, 23. Consistent with these reports, our results showed high expression of PVT1 in the PDAC cancer and PVT1 could facilitate glycolysis and tumor progression. Part of miRNAs has been regarded as a key regulator of glycolysis inhibition and tumor progression. Several previous reports demonstrated that miR-519d-3p is downregulated in many cancers and could inhibit cell proliferation and migration 13, 14.

PVT1 functions as a molecular sponge to competitively inhibit miRNAs, including miR-448, miR-195, miR-497, miR-20a-5p 6, 9, 12,20,23. PVT1 has been reported to sponge miR-519d-3p to facilitate invasion and proliferation in laryngeal squamous cell cancer^15^. We speculated that PVT1 may facilitate PDAC progression and glycolysis through down regulation of miR-519d-3p.

The results from the luciferase reporter assay further supported direct interaction between PVT1 and miR-519d-3p. Furthermore, rescue experiments showed that miR-519d-3p inhibitor completely reversed the inhibition of glycolysis, cell proliferation and invasion ability induced by PVT1 knockdown.

HIF-1 play a role in in angiogenesis, glucose metabolism, cell proliferation/survival and invasion/ metastasis by activating the transcription of many genes. 24. Previous research revealed that HIF-1 is invariably upregulated in PDAC 25. Since PVT1/ miR-519d-3p and HIF-1A participate in regulating PDAC cancer progression and glycolysis, we researched whether PVT1/ miR-519d-3p functions through regulating HIF-1A. The luciferase assay revealed that miR-519d-3p could be in direct interaction with the 3'UTR mRNA of HIF-1A and negatively regulate its expression. Tumor xenografts experiments revealed that PVT1 level correlated with miR-519d-3p and HIF-1A level. In summary, these showed that PVT1 produced an oncogenic role in PDAC partly by miR-519d-3p/HIF-1A.

## Figures and Tables

**Table 1:** Correlation of PVT-1 expression to clinicopathologic features in PDAC

	PVT1		*Χ*^2^	*P*
	Low	High		
**Sex**			2.143	0.143
Male	9	6		
Female	5	10		
**Age**				
<65	10	10	0.268	0.605
≥65	4	6		
**Lymph node metastasis**			8.438	0.004*
No	10	3		
Yes	4	13		
**pTNM stage**			3.846	0.050
I+II	13	10		
III+IV	1	6		
**Differentiation**				
Well	5	1	4.152	0.125
Moderate	6	9		
Poor	3	6		
**Tumor size(cm)**			3.772	0.052
<3	11	7		
≥3	3	9		

Values of *P* were calculated by the Spearman's rank-correlation test. pTNM stage refers to the pathological tumor node metastasis (pTNM) stage designed jointly by the UICC (Union Internationale Against Cancer) and the AJCC (American Joint Committee on Cancer) in 2018. * Statistically significant (P < 0.05).
